# A PINN-driven game-theoretic framework in limited data photoacoustic tomography

**DOI:** 10.1088/1361-6420/ae1bcd

**Published:** 2025-11-14

**Authors:** Souvik Roy, Suvra Pal

**Affiliations:** 1 Department of Mathematics, The University of Texas at Arlington, Arlington, TX 76019-0407, United States of America; 2 Division of Data Science, College of Science, The University of Texas at Arlington, Arlington, TX 76019-0407, United States of America

**Keywords:** Nash equilibrium, machine learning, sequential quadratic Hamiltonian, Pontryagin’s maximum principle, tomographic imaging

## Abstract

This paper presents a novel methodological framework to obtain superior reconstructions in limited data photoacoustic tomography. The proposed framework exploits the presence of Cauchy data on an accessible part of the observation domain and uses a Nash game-theoretic framework to complete the missing data on the inaccessible region. To solve the game-theoretic problem, a gradient-free sequential quadratic Hamiltonian scheme, which is based on Pontryagin’s maximum principle characterization, is combined with physics-informed neural networks to obtain the initial guess, leading to a robust and accurate reconstruction scheme. Numerical simulations with various phantoms, choice of accessible observation domains, and noise, demonstrate the effectiveness of our proposed framework to obtain high contrast and resolution reconstructions.

## 1. Introduction

Optical tomography (OT) is an imaging technique that uses light propagation in the near-infrared spectral region to reconstruct the optical properties of biological objects such as tissues. Since cancerous tissues exhibit low light scattering in comparison to healthy tissues, accurate reconstructions of the scattering and the absorption properties of tissues help in a proper diagnosis of cancer. However, reconstructions in OT are highly ill-posed and suffer from a huge loss of resolution; thus, to obtain high-fidelity optical properties, several coupled-physics modalities, like fluorescence molecular tomography, confocal diffuse tomography, single-photon photoacoustic tomography, two-photon photoacoustic tomography, ultrasound modulated OT, have been developed in OT and are known as hybrid OT (see e.g. [14]). One of the most popular hybrid OT modalities is the photoacoustic tomography (PAT) [12], which finds important applications in biomedical studies. Thus, computational models for obtaining accurate reconstructions in PAT are of paramount importance.

In PAT, an object of interest, that is typically heterogeneous, such as a biological tissue, is irradiated with short-pulse near-infrared (NIR) light along its boundary. The object absorbs a portion of the photons during this process, which leads to a rise in the temperature of the object and results in its thermal expansion, and the remaining photons are scattered. After the exit of the remaining photons, the object cools down and contracts. This phenomenon of successive expansion and contraction leads to a pressure change inside the object, which then propagates through the object as acoustic or ultrasound waves, and is also known as the photoacoustic effect. The acoustic wave pressure is then measured by ultrasound sensors placed on the boundary of the object. Thus, the propagation of the acoustic waves occurs on a scale of microseconds that is about three orders of magnitude lower than the optical propagation; this is the mathematical problem of reconstructing optical properties in PAT. The inverse problem is to recover the initial acoustic wave pressure distribution, also known as the interior optical energy density data, from the measured acoustic wave pressure.

A major challenge for developing PAT reconstruction algorithms is the limited availability of measurements in the acquisition domain. One of the main reasons for this is the involvement of higher cost and time for obtaining data at all points in the acquisition domain. Furthermore, some of the regions in the acquisition domain might not be accessible for data measurements, e.g. due to the presence of pacemakers, a broken spine, or near heart valves. There are works that have studied the theoretical properties of the limited PAT problem [32] and also provided algorithms, based on the Neumann series expansion [27] and time-reversal (TR) methods [17], for obtaining numerical reconstructions. This issue of limited data measurements usually leads to the presence of severe artifacts in the reconstructions with both classical inversion and variational methods [3, 13].

Existing computational algorithms in PAT do not make use of additional available data on the accessible measurement boundary that actually provides important information about the data on the inaccessible part. In [22], it has been shown that the flux measurements (representing Neumann data) can also be computed in the acquisition subdomain along with the acoustic pressure measurements (representing Dirichlet data). In [2], the Neumann data was obtained as a function of the time derivative of the Dirichlet data based on the impedance of the ultrasound sensors, In [23, 25, 31], experimental setups for breast mammography in functional PAT were used to record acoustic pressure measurements on a ring of ultrasound transducers with varying radii. Using such measurements one can compute the flux measurements. In this work, we aim to exploit these crucial observations and results to develop a new computational algorithm that can first complete the acoustic wave data on the missing part of the boundary, with the help of the additional acoustic flux measurements, and then solve for the initial pressure source. This is called the simultaneous data completion and identification inverse problem. Several methods for data completion have been proposed based on sampling techniques, like level set approaches [7], shape optimization methods [8], optimal control formulations [1], iterative Tikhonov regularization [10]. However, none of these works deal with highly ill-posed inverse problems arising in tomographic imaging.

Game-theoretic models provide several robust frameworks for solving ill-posed dynamical and inverse problems. One such framework is the differential game that involves several players governed by an underlying differential model, which represents the state of the underlying system. A differential game is subject to various actions, represented as strategies, of the players in the game. Each player has an associated objective functional and admissible set of actions, and the purpose of the game is for each player to minimize its objective subject to the constraints given by the differential model, with strategies obtained from the admissible sets. For non-cooperative games, an appropriate solution concept, known as the equilibrium solution, is the one proposed by Nash in [24], where a Nash equilibrium (NE) for static games with complete information is defined. In this configuration, no player can benefit from unilaterally changing its own strategy. Such a differential Nash game-theoretic framework was pioneered by Isaacs in [5]. Subsequently, Nash-game frameworks were considered by several authors for numerous applications arising in marketing, economics and avoidance phenomenon (e.g. see [30]).

The Nash game-theoretic framework provides a natural extension of traditional variational formulations by decomposing the global data completion problem into two coupled subproblems associated with the Dirichlet and Neumann data. Each subproblem, represented as a ‘player’, minimizes its own cost functional while interacting through a coupling term that enforces mutual consistency of the reconstructed field. This structure enhances numerical stability, introduces an inherent regularization effect, and enables efficient implementation using gradient-free schemes such as the sequential quadratic Hamiltonian (SQH) method. While other hierarchical game models (e.g. Stackelberg games) could be considered when one data type is dominant, the symmetric Nash formulation is particularly suitable for PAT, where both pressure and flux data play equally significant roles.

In the context of data-completion, a Nash-game framework was first studied by the authors in [15, 16] for elliptic partial differential equations (PDEs). Subsequently, in [21], the authors propose a Nash-game framework to solve a problem of image inpainting via data completion. In [20], the authors solve a coupled inverse problem that combines data completion and the determination of the best locations of an unknown number of small objects immersed in a stationary viscous fluid. However, game-theoretic frameworks have rarely been used for developing computational algorithms to solve inverse problems. Very recently, in [9], the authors solved a coupled data completion and reconstruction of conductivity in electrical impedance tomography (EIT). Even though the results, obtained in [9], are robust and accurate for the limited data problem in EIT, where the underlying PDE is elliptic, the Nash games framework remains unexplored for other tomographic inverse problems, especially, for the ones where the dynamics are governed by hyperbolic PDEs, like in PAT.

We develop a novel computational scheme using a Nash games framework that will enable us to first complete the Dirichlet acoustic pressure data on the inaccessible part of the acquisition domain using Cauchy data on the accessible part, and then reconstruct the initial acoustic pressure source, employing a iterative and gradient-free SQH scheme, based on the Pontryagin’s maximum principle. However, the performance of such iterative schemes usually depend on the initial guess, which poses a significant challenge for a robust and stable reconstruction process. To encounter this issue, we use the framework of physics-informed neural networks (PINNs), which can handle sparse and noisy datasets to yield robust parameter estimates [28, 35]. PINNs integrate the governing wave equation, boundary conditions, and initial conditions into their loss function, enabling them to incorporate the underlying physics directly into the solution process. This eliminates the need for extensive datasets, as the physics itself acts as a regularizer. PINNs are highly flexible and can handle complex, high-dimensional PDEs where traditional numerical methods struggle due to challenges like computational cost or mesh generation. With their reliance on automatic differentiation, PINNs offer end-to-end differentiability, making it straightforward to compute gradients with respect to unknown parameters, which is crucial for inverse problems. Moreover, they can efficiently handle sparse and noisy data by combining physical constraints with limited measurements, making them robust in real-world applications [33]. We combine the SQH scheme with the PINN solver to obtain a robust, accurate, and fast implementation of the simultaneous data completion and identification problem.

The paper is organized as follows: In the next section, we describe a Nash game-theoretic framework for data completion and initial acoustic source identification. Section 3 deals with the theoretical results about the game-theoretic problem. We describe the SQH and PINN frameworks to numerically implement the game-theoretic problem in section 4. Numerical results with synthetic phantoms are presented in section 5 along with a comprehensive comparison with varying the accessible observation domains and different types of noise levels. A section on conclusion ends the work.

## 2. Problem formulation

We consider the model of wave propagation in PAT as follows

\begin{align*} p_{tt}-c\left(x\right)\Delta p\left(x,t\right) &amp; = 0, \qquad \mbox{in } \Omega \times \left(0,T\right), \nonumber\\ p\left(x,0\right) &amp; = p_0\left(x\right), \qquad \mbox{in } \Omega, \nonumber\\ p_t\left(x,0\right) &amp; = 0, \qquad \mbox{in } \Omega, \end{align*}

where $x\in \Omega \subset \mathbb{R}^n$, $n\unicode{x2A7E} 1$, $p(x,t)$ is the pressure of the acoustic wave at the point (*x*, *t*) with the unknown initial pressure field as $p_0(x)$, and *c*(*x*) is the sound speed. Let $\partial \Omega_a$ be the accessible part of the observation domain $\partial\Omega$, where the acoustic pressure data $p_d(x,t)$ and the acoustic flux data $p_n (x,t)$ is collected. The acoustic pressure data represents the instantaneous pressure amplitude exerted by the propagating wave on the sensor surface and corresponds to the Dirichlet boundary data of the wave equation. In contrast, the acoustic flux data denotes the normal derivative of the pressure, which characterizes the rate of momentum or energy flow across the sensor surface and corresponds to the Neumann boundary data. Denote $\partial\Omega_i = \partial\Omega \setminus \partial \Omega_a$ as the inaccessible part of the observation domain $\partial\Omega$. The inverse problem is to determine *p*_0_ from $p_d,p_n$. The traditional time reversal algorithm solves the following Cauchy PDE [19]:

\begin{align*} \begin{aligned} q_{tt}-c\left(x\right)\Delta q\left(x,t\right) = 0 &amp;, \qquad \mbox{in } \Omega \times \left(0,T\right),\\ q\left(x,0\right) = 0 &amp;, \qquad \mbox{in } \Omega,\\ q_t\left(x,0\right) = 0 &amp;, \qquad \mbox{in } \Omega,\\ q\left(x,t\right) = p_d\left(x,T-t\right) &amp;, \qquad \mbox{on } \partial\Omega_a \times \left(0,T\right),\\ q\left(x,t\right) = 0 &amp;, \qquad \mbox{on } \partial\Omega_i \times \left(0,T\right).\\ \end{aligned}\end{align*}

Then $p_0(x) = q(x,T)$. Several works discuss the solution of the aforementioned TR algorithm for the limited data case [17, 18]. However, it has been observed that the reconstructions obtained suffer from the presence of severe artifacts, even in the case of non-trapping sound speed *c*. We note that the aforementioned works do not take into account the information of the flux data *p_n_* on the accessible part of the boundary.

We, thus, employ an alternate technique that uses both $p_d,p_n$ for superior reconstructions in PAT. Using the idea of the TR method, we consider the following Cauchy PDE:

\begin{align*} \begin{aligned} &amp;q_{tt}-c\left(x\right)\Delta q\left(x,t\right) = 0, ~ \mbox{in } \Omega \times \left(0,T\right),\\ &amp;q\left(x,0\right) = 0, ~ \mbox{in } \Omega,\\ &amp;q_t\left(x,0\right) = 0, ~ \mbox{in } \Omega,\\ &amp;q\left(x,t\right) = p_d\left(x,T-t\right), ~ \mbox{on } \partial\Omega_a \times \left(0,T\right),\\ &amp;\dfrac{\partial q}{\partial n}\left(x,t\right) = p_n\left(x,T-t\right),~ \mbox{on } \partial\Omega_a \times \left(0,T\right).\\ \end{aligned}\end{align*}

We refer to this PDE as the limited data Cauchy problem. Our goal is to use the Cauchy data $p_n,p_d \in C^0([0,T]; H^{\mp 1/2}(\partial\Omega_a))$ to determine the missing boundary data and then solve (3). For this purpose, we consider the following Cauchy problems:

\begin{align*} \begin{aligned} q^1_{tt}-c\left(x\right)\Delta q^1\left(x,t\right) = 0 &amp;, \qquad \mbox{in } \Omega \times \left(0,T\right),\\ q^1\left(x,0\right) = 0 &amp;, \qquad \mbox{in } \Omega,\\ q^1_t\left(x,0\right) = 0 &amp;, \qquad \mbox{in } \Omega,\\ q^1\left(x,t\right) = p_d\left(x,T-t\right) &amp;, \qquad \mbox{on } \partial\Omega_a \times \left(0,T\right),\\ \dfrac{\partial q^1}{\partial n}\left(x,t\right) = \eta &amp;, \qquad \mbox{on } \partial\Omega_i \times \left(0,T\right),\\ \end{aligned}\end{align*}

\begin{align*} \begin{aligned} q^2_{tt}-c\left(x\right)\Delta q^2\left(x,t\right) = 0 &amp;, \qquad \mbox{in } \Omega \times \left(0,T\right),\\ q^2\left(x,0\right) = 0 &amp;, \qquad \mbox{in } \Omega,\\ q^2_t\left(x,0\right) = 0 &amp;, \qquad \mbox{in } \Omega,\\ q^2\left(x,t\right) = \tau &amp;, \qquad \mbox{on } \partial\Omega_i \times \left(0,T\right),\\ \dfrac{\partial q^2}{\partial n}\left(x,t\right) = p_n\left(x,T-t\right) &amp;, \qquad \mbox{on } \partial\Omega_a \times \left(0,T\right),\\ \end{aligned}\end{align*}

where $\eta,\tau \in C^0([0,T]; H^{\mp 1/2}(\partial\Omega_i))$ are unknown functions to be determined, also termed as the missing data. We denote the equations (4) and (5) as $\mathcal{L}(q^1,p_d,\eta) = 0$ and $\mathcal{L}(q^2,p_n,\tau) = 0$, respectively. To determine $\eta,\tau$, we formulate a data completion framework for solving inverse problems related to hyperbolic wave equations in the realm of differential game theory. Our game consists of two players and can be formulated as follows: Player 1 has control over the strategy *η* with its associated cost functional given as

\begin{align*} J_1\left(\eta,\tau\right) = \dfrac{\alpha_1}{2} \int_0^T\int_{\partial\Omega_a}\left(\dfrac{\partial q^1}{\partial n} -p_n\right)^2dsdt + \dfrac{w}{2} \int_0^T \int_{\Omega} \left(q^1-q^2\right)^2dxdt.\end{align*}

Player 2 has a control over the strategy *τ* with its associated cost functional given as

\begin{align*} J_2\left(\eta,\tau\right) = \dfrac{\alpha_2}{2} \int_0^T\int_{\partial\Omega_a}\left(q^2 -p_d\right)^2dsdt + \dfrac{w}{2} \int_0^T \int_{\Omega} \left(q^1-q^2\right)^2dxdt.\end{align*}

We desire for a Nash equilibrium (NE) of the game described above, i.e. we need to determine $ S_w^* = (\eta^*,\tau^*)\in C^0([0,T]; H^{\mp1/2}(\partial\Omega_i)) $ such that $S_w^*$ is a Nash equilibrium (NE) for the game, i.e. $S_w^*$ satisfies

\begin{align*} \begin{aligned} &amp;J_1\left(\eta^*,\tau^*\right) \unicode{x2A7D} J_1\left(\eta,\tau^*\right), ~ J_2\left(\eta^*,\tau^*\right) \unicode{x2A7D} J_2\left(\eta^*,\tau\right),~\forall \eta,\tau \in S^{\mp} : = C^0\left(\left[0,T\right]; H^{\mp 1/2}\left(\partial\Omega_i\right)\right). \end{aligned}\end{align*}

We refer to (6)–(8) as our NASH GAME.

## 3. Theoretical results

In this section, we discuss some theoretical properties of our NASH GAME. We start with the well-posedness result for the limited data Cauchy problem (3) whose proof is given in [4].

Proposition 1 (Well-posedness; Bardos, Lebau, Rauch’92 ). The limited data Cauchy problem (3) has a well-posed solution, i.e. the boundary measurements $p_d,p_n$ uniquely determine the solution *q*, if *T* is large enough ($ > rad(\bar{\Omega})/\min(c))$ such that every generalized bicharacteristic of the wave operator intersects $\partial\Omega_a$ at a non-diffractive point (geometric control condition).

The aforementioned result implies that for a final time *T* satisfying the geometric control condition, some acoustic waves will always be recorded in an acquisition domain on a part of $\partial \Omega$ as long as the waves are not trapped entirely in the inaccessible domain. This can be achieved with even irregular geometries of Ω, specifically when Ω has a combination of different polygonal boundaries, with detectors lying only on one of the flat polygonal edges.

We next show the well-posedness of our NASH GAME problem.

Theorem 2 (Optimal control problem). A pair $(\eta^*,\tau^*)$ is a solution to the NASH GAME iff it is a solution to the following optimal control problem

\begin{align*} \min_{\eta,\tau} J\left(\eta,\tau\right) = &amp; \dfrac{\alpha_1}{2} \int_0^T\int_{\partial\Omega_a}\left(\dfrac{\partial q^1}{\partial n} -p_n\right)^2dsdt +\dfrac{\alpha_2}{2} \int_0^T\int_{\partial\Omega_a}\left(q^2 -p_d\right)^2dsdt\nonumber\\ &amp; +\dfrac{w}{2} \int_0^T \int_{\Omega} \left(q^1-q^2\right)^2dxdt \end{align*}

Proof. Let $(\eta^*,\tau^*)$ be a solution to the optimal control problem (9). Then $J(\eta^*,\tau^*) \unicode{x2A7D} J(\eta,\tau),~\forall \eta,\tau \in S^{\mp} $. Fixing $\tau = \tau^*$, we have

\begin{equation*} J\left(\eta^*,\tau^*\right) \unicode{x2A7D} J\left(\eta,\tau^*\right),\end{equation*}

which implies

\begin{align*} &amp;\dfrac{\alpha_2}{2} \int_0^T\int_{\partial\Omega_a}\left(q^2_* -p_d\right)^2dsdt + \dfrac{\alpha_1}{2} \int_0^T\int_{\partial\Omega_a}\left(\dfrac{\partial q^1_*}{\partial n} -p_n\right)^2dsdt +\dfrac{w}{2} \int_0^T \int_{\Omega} \left(q^1_*-q^2_*\right)^2dxdt\\ &amp;\quad \unicode{x2A7D} \dfrac{\alpha_2}{2} \int_0^T\int_{\partial\Omega_a}\left(q^2 -p_d\right)^2dsdt + \dfrac{\alpha_1}{2} \int_0^T\int_{\partial\Omega_a}\left(\dfrac{\partial q^1_*}{\partial n} -p_n\right)^2dsdt +\dfrac{w}{2} \int_0^T \int_{\Omega} \left(q^1-q^2_*\right)^2dxdt. \end{align*}

This gives us

\begin{align*} &amp;\dfrac{\alpha_2}{2} \int_0^T\int_{\partial\Omega_a}\left(q^2_* -p_d\right)^2dsdt +\dfrac{w}{2} \int_0^T \int_{\Omega} \left(q^1_*-q^2_*\right)^2dxdt\\ &amp;\quad \unicode{x2A7D} \dfrac{\alpha_2}{2} \int_0^T\int_{\partial\Omega_a}\left(q^2 -p_d\right)^2dsdt +\dfrac{w}{2} \int_0^T \int_{\Omega} \left(q^1_*-q^2\right)^2dxdt, \end{align*}

which implies

\begin{equation*} J_2\left(\eta^*,\tau^*\right) \unicode{x2A7D} J_2\left(\eta^*,\tau\right).\end{equation*}

In a similar way, we have $J_1(\eta^*,\tau^*) \unicode{x2A7D} J_1(\eta,\tau^*)$ and, thus, $(\eta^*,\tau^*)$ is a NE of the NASH GAME.

Conversely, let $(\eta^*,\tau^*)$ be an NE of the NASH GAME. Then we have

\begin{equation*} J_1\left(\eta^*,\tau^*\right) \unicode{x2A7D} J_1\left(\eta,\tau^*\right), ~ J_2\left(\eta^*,\tau^*\right) \unicode{x2A7D} J_2\left(\eta^*,\tau\right).\end{equation*}

Adding $\dfrac{\alpha_2}{2} \int_0^T\int_{\partial\Omega_a}(q^2 -p_d)^2dsdt $ to the first inequality and $\dfrac{\alpha_1}{2}\int_0^T\int_{\partial\Omega_a}\left(\dfrac{\partial q^1}{\partial n} -p_n\right)^2dsdt $ to the second one, we obtain

\begin{equation*} J\left(\eta^*,\tau^*\right) \unicode{x2A7D} J\left(\eta,\tau^*\right), ~ J\left(\eta^*,\tau^*\right) \unicode{x2A7D} J\left(\eta^*,\tau\right),~ \forall \left(\eta,\tau\right) \in S_{\mp}.\end{equation*}

This gives us

\begin{equation*} \dfrac{\partial J}{\partial \eta}\left(\eta^*,\tau^*\right) = 0,~\dfrac{\partial J}{\partial \tau}\left(\eta^*,\tau^*\right) = 0,\end{equation*}

which are the optimality conditions for the optimal control problem (9). Thus, we have $(\eta^*,\tau^*)$ as an optimal control of (9). □

Proposition 3 (Existence and uniqueness of NE). Given the Cauchy data $p_n,p_d \in C^0([0,T]; H^{\mp 1/2}(\partial\Omega_a))$, there exists an unique Nash equilibrium $(\eta^*,\tau^*)\in S^{\mp} $ for the NASH GAME.

Proof. The functionals $J_1,J_2$ are quadratic in $\eta,\tau$, respectively, since $q^1,q^2$ depends linearly on $\eta,\tau$, respectively. The second-order differential of *J*_1_ with respect to *η* in the direction $\psi \in L^2(\Gamma_i)$ is given by:

\begin{equation*}\langle D^2 J_1\left(\eta, \tau\right) \left(\psi\right), \psi\rangle = \alpha_1\left\|\dfrac{\partial q^1_0}{\partial n}\left(\psi\right)\right\|^2_{L^2\left(\partial\Omega_a\right)}+w\|q^1_0\left(\psi\right)\|^2_{L^2\left(\Omega\right)},\end{equation*}

and the second-order differential of *J*_2_ with respect to *τ* in the direction $\psi \in H^{1/2}(\Gamma_i)$ is given by:

\begin{equation*}\langle D^2 J_2\left(\eta, \tau\right) \left(\psi\right), \psi\rangle = \alpha_2\|q^2_0\left(\psi\right)\|^2_{L^2\left(\partial\Omega_a\right)} +w\|q^2_0\left(\psi\right)\|^2_{L^2\left(\Omega\right)},\end{equation*}

where $q^1_0(\psi),q^2_0(\psi)$ solves

\begin{align*} \begin{aligned} \left(q^1_0\right)_{tt}-c\left(x\right)\Delta q^1_0\left(x,t\right) &amp; = 0, \qquad \mbox{in } \Omega \times \left(0,T\right),\\ q^1_0\left(x,0\right) &amp; = 0, \qquad \mbox{in } \Omega,\\ \left(q^1_0\right)_t\left(x,0\right) &amp; = 0, \qquad \mbox{in } \Omega,\\ q^1_0\left(x,t\right) &amp; = 0, \qquad \mbox{on } \partial\Omega_a \times \left(0,T\right),\\ \dfrac{\partial q^1_0}{\partial n}\left(x,t\right) &amp; = \psi, \qquad \mbox{on } \partial\Omega_i \times \left(0,T\right),\\ \end{aligned}\end{align*}

\begin{align*} \begin{aligned} \left(q^2_0\right)_{tt}-c\left(x\right)\Delta q^2_0\left(x,t\right) &amp; = 0, \qquad \mbox{in } \Omega \times \left(0,T\right),\\ q^2_0\left(x,0\right) &amp; = 0, \qquad \mbox{in } \Omega,\\ \left(q^2_0\right)_t\left(x,0\right) &amp; = 0, \qquad \mbox{in } \Omega,\\ q^2_0\left(x,t\right) &amp; = \psi, \qquad \mbox{on } \partial\Omega_i \times \left(0,T\right),\\ \dfrac{\partial q^2_0}{\partial n}\left(x,t\right) &amp; = 0, \qquad \mbox{on } \partial\Omega_a \times \left(0,T\right).\\ \end{aligned}\end{align*}

We note that if *ψ* ≠ 0, $\langle D^2 J_i(\eta, \tau) (\psi), \psi\rangle > 0$ which implies that $J_1,J_2$ are strictly convex with respect to $\eta,\tau$, respectively. Furthermore, both $J_1(\eta, \tau)$ and $J_2(\eta, \tau)$ are continuous with respect to both *η* and *τ*, and coercive with respect to $\eta,\tau$, respectively.

Define the best-response maps as follows:

\begin{align*} \begin{aligned} \mathcal{R}_\eta\left(\tau\right)&amp; = \left\{\eta\in S^-:\eta = \mathrm{arg\,min}_{S^-} J_1\left(\eta,\tau\right) \right\}\\ \mathcal{R}_\tau\left(\eta\right)&amp; = \left\{\tau\in S^+:\tau = \mathrm{arg\,min}_{S^+} J_2\left(\eta,\tau\right) \right\}. \end{aligned}\end{align*}

Then a NE of the NASH GAME is given as:

\begin{equation*} \eta^* = \mathcal{R}_\eta\left(\tau^*\right),~ \tau^* = \mathcal{R}_\tau\left(\eta^*\right).\end{equation*}

With this preparation, we define the mapping

\begin{equation*} \mathcal{T}: S^-\times S^+ \rightarrow S^-\times S^+,~ \mathcal{T}\left(\eta,\tau\right) = \left(\mathcal{R}_\eta\left(\tau\right),\mathcal{R}_\tau\left(\eta\right)\right).\end{equation*}

Then the coercivity of $J_1,J_2$ implies that one can choose a large enough closed bounded balls $B_1,B_2$ such that $B = B_1\times B_2$ is a weakly compact convex subsets of the Hilbert space $S^-\times S^+$ and $\mathcal{T}(B)\subset B$. Furthermore, continuity and convexity of $J_1,J_2$ implies weak lower semicontinuity of $J_1,J_2$ over $B_1,B_2$. Thus, $\mathcal{T}:B\rightarrow B$ satisfies all the conditions of Schauder’s fixed point theorem and thus has at least one fixed point $(\eta^*,\tau^*)$ which is an NE of the NASH GAME.

For the uniqueness, note that the strict convexity of $J_1,J_2$ implies the strict convexity of *J* with respect to $(\eta,\tau)$. Thus, *J* has an unique optimal control $(\eta^*,\tau^*)$, which implies that the NE is unique. □

Theorem 4 (Data completion). The NE $(\eta^*,\tau^*)$ is the missing data of the Cauchy PDE (3), i.e. $q(x,t) = \tau^*,~ \dfrac{\partial q}{\partial n}(x,t) = \eta^*$ on $\partial \Omega_i \times (0,T).$

Proof. Let *q* be the unique solution of (3) and let $\tilde{\eta} = \dfrac{\partial q}{\partial n},~ \tilde{\tau} = q $ on $\partial\Omega_i$. Then $q^1 = q^2 = q$ in Ω, $\dfrac{\partial q^1}{\partial n} = p_n$ on $\partial\Omega_a$, and $q^2 = p_d$ on $\partial\Omega_a$. This gives us $J_1(\tilde{\eta},\tilde{\tau}) = 0 = J_2(\tilde{\eta},\tilde{\tau})$, which implies that $(\tilde{\eta},\tilde{\tau})$ is a NE of the NASH GAME. Since the NE is unique, we have that $(\eta^*,\tau^*) = (\tilde{\eta},\tilde{\tau}) = \left(\dfrac{\partial q}{\partial n},q\right)$ on $\partial\Omega_i$, which is our missing data of the Cauchy PDE (3). □

We now consider the case of noisy data. For this purpose, we consider the noisy data pair $p^\delta_n,p^\delta_d \in C^0([0,T]; H^{\mp 1/2}(\partial\Omega_a))$ satisfying

\begin{equation*} \|p^\delta_d-p_d\|_{C^0\left(\left[0,T\right]; H^{1/2}\left(\partial\Omega_a\right)\right)}^2+ \|p^\delta_n-p_n\|_{C^0\left(\left[0,T\right]; H^{-1/2}\left(\partial\Omega_a\right)\right)}^2 < \delta^2.\end{equation*}

The corresponding cost functionals for noisy data are given by:

\begin{equation*} J^\delta_1\left(\eta, \tau\right) = \dfrac{\alpha_1}{2} \int_0^T\int_{\partial\Omega_a}\left(\dfrac{\partial q^1_\delta}{\partial n} -p_n^\delta\right)^2dsdt + \dfrac{w}{2} \int_0^T \int_{\Omega} \left(q^1_\delta-q^2_\delta\right)^2dxdt,\end{equation*}

\begin{equation*} J^\delta_2\left(\eta, \tau\right) = \dfrac{\alpha_2}{2} \int_0^T\int_{\partial\Omega_a}\left(q^2_\delta -p_d^\delta\right)^2dsdt + \dfrac{w}{2} \int_0^T \int_{\Omega} \left(q^1_\delta-q^2_\delta\right)^2dxdt,\end{equation*}

where $q^1_\delta$ and $q^2_\delta$ are solutions to the perturbed boundary value problems:

\begin{align*} \begin{aligned} \left(q^1_\delta\right)_{tt}-c\left(x\right)\Delta q^1_\delta\left(x,t\right) = 0 &amp;, \qquad \mbox{in } \Omega \times \left(0,T\right),\\ q^1_\delta\left(x,0\right) = 0 &amp;, \qquad \mbox{in } \Omega,\\ \left(q^1_\delta\right)_t\left(x,0\right) = 0 &amp;, \qquad \mbox{in } \Omega,\\ q^1_\delta\left(x,t\right) = p_d^\delta\left(x,T-t\right) &amp;, \qquad \mbox{on } \partial\Omega_a \times \left(0,T\right),\\ \dfrac{\partial q^1_\delta}{\partial n}\left(x,t\right) = \eta &amp;, \qquad \mbox{on } \partial\Omega_i \times \left(0,T\right),\\ \end{aligned}\end{align*}

\begin{align*} \begin{aligned} \left(q^2_\delta\right)_{tt}-c\left(x\right)\Delta q^2_\delta\left(x,t\right) = 0 &amp;, \qquad \mbox{in } \Omega \times \left(0,T\right),\\ q^2_\delta\left(x,0\right) = 0 &amp;, \qquad \mbox{in } \Omega,\\ \left(q^2_\delta\right)_t\left(x,0\right) = 0 &amp;, \qquad \mbox{in } \Omega,\\ q^2_\delta\left(x,t\right) = \tau &amp;, \qquad \mbox{on } \partial\Omega_i \times \left(0,T\right),\\ \dfrac{\partial q^2_\delta}{\partial n}\left(x,t\right) = p_n^\delta\left(x,T-t\right) &amp;, \qquad \mbox{on } \partial\Omega_a \times \left(0,T\right).\\ \end{aligned}\end{align*}

We have the following stability and convergence result:

Theorem 5 (Noisy data stability). Let the limited data Cauchy problem have a solution $q\in C^0([0,T];H^1(\Omega))$ and let $p^\delta_n,p^\delta_d \in C^0([0,T]; H^{\mp 1/2}(\partial\Omega_a))$ be such that

\begin{equation*} \|p^\delta_d-p_d\|_{C^0\left(\left[0,T\right]; H^{1/2}\left(\partial\Omega_a\right)\right)}^2+ \|p^\delta_n-p_n\|_{C^0\left(\left[0,T\right]; H^{-1/2}\left(\partial\Omega_a\right)\right)}^2 < \delta^2.\end{equation*}

Then the NASH GAME with the modified costs involving $p^\delta_d,p^\delta_n$ has an unique Nash equilibrium $(\eta^*_\delta,\tau^*_\delta)$ such that as $\delta \rightarrow 0$

\begin{equation*} \|\eta^*_\delta-\eta^*\|_{C^0\left(\left[0,T\right]; H^{-1/2}\left(\partial\Omega_i\right)\right)}\rightarrow 0,~ \|\tau^*_\delta-\tau^*\|_{C^0\left(\left[0,T\right]; H^{1/2}\left(\partial\Omega_i\right)\right)}\rightarrow 0,\end{equation*}

and

\begin{equation*} \|q^1_\delta-q\|_{C^0\left(\left[0,T\right]; H^{1}\left(\Omega\right)\right)}\rightarrow 0,~ \|q^2_\delta-q\|_{C^0\left(\left[0,T\right]; H^{1}\left(\Omega\right)\right)}\rightarrow 0.\end{equation*}

Proof. By proposition 3, the perturbed functionals $J^\delta_1$ and $J^\delta_2$ are quadratic, coercive, and strictly convex with respect to *η* and *τ*, respectively. Using the Schauder fixed-point theorem, we have the existence of an unique Nash equilibrium $(\eta^*_\delta, \tau^*_\delta)$. We now define the following auxiliary variables $z^1_\delta = q^1_\delta(\eta^*) - q$ and $z^2_\delta = q^2_\delta(\tau^*) - q$, where $(\eta^*, \tau^*)$ are the true missing data. Then, $z^1_\delta$ and $z^2_\delta$ solve the following perturbation problems:

\begin{align*} \begin{aligned} \left(z^1_\delta\right)_{tt}-c\left(x\right)\Delta z^1_\delta\left(x,t\right) = 0 &amp;, \qquad \mbox{in } \Omega \times \left(0,T\right),\\ z^1_\delta\left(x,0\right) = 0 &amp;, \qquad \mbox{in } \Omega,\\ \left(z^1_\delta\right)_t\left(x,0\right) = 0 &amp;, \qquad \mbox{in } \Omega,\\ z^1_\delta\left(x,t\right) = p_d^\delta\left(x,T-t\right)-p_d\left(x,T-t\right) &amp;, \qquad \mbox{on } \partial\Omega_a \times \left(0,T\right),\\ \dfrac{\partial z^1_\delta}{\partial n}\left(x,t\right) = 0 &amp;, \qquad \mbox{on } \partial\Omega_i \times \left(0,T\right),\\ \end{aligned}\end{align*}

and

\begin{align*} \begin{aligned} \left(z^2_\delta\right)_{tt}-c\left(x\right)\Delta z^2_\delta\left(x,t\right) = 0 &amp;, \qquad \mbox{in } \Omega \times \left(0,T\right),\\ z^2_\delta\left(x,0\right) = 0 &amp;, \qquad \mbox{in } \Omega,\\ \left(z^2_\delta\right)_t\left(x,0\right) = 0 &amp;, \qquad \mbox{in } \Omega,\\ z^2_\delta\left(x,t\right) = 0 &amp;, \qquad \mbox{on } \partial\Omega_i \times \left(0,T\right),\\ \dfrac{\partial z^2_\delta}{\partial n}\left(x,t\right) = p_n^\delta\left(x,T-t\right)-p_n\left(x,T-t\right) &amp;, \qquad \mbox{on } \partial\Omega_a \times \left(0,T\right).\\ \end{aligned}\end{align*}

The perturbed functional $ J^\delta(\eta, \tau) $ is defined as:

\begin{align*} J^\delta\left(\eta, \tau\right) &amp; = \dfrac{\alpha_1}{2} \int_0^T\int_{\partial\Omega_a}\left(\dfrac{\partial q^1_\delta}{\partial n} -p_n^\delta\right)^2dsdt \\ &amp;\quad + \dfrac{\alpha_2}{2} \int_0^T\int_{\partial\Omega_a}\left(q^2_\delta -p_d^\delta\right)^2dsdt + \dfrac{w}{2} \int_0^T \int_{\Omega} \left(q^1_\delta-q^2_\delta\right)^2dxdt.\end{align*}

Substituting $ q^1_\delta(\eta^*) = z^1_\delta + q $ and $ q^2_\delta(\tau^*) = z^2_\delta + q $, we obtain:

\begin{align*} J^\delta\left(\eta^*, \tau^*\right) &amp; = \dfrac{\alpha_1}{2} \int_0^T\int_{\partial\Omega_a}\left(\dfrac{\partial q^1_\delta}{\partial n} -\dfrac{\partial q^2_\delta}{\partial n}\right)^2dsdt \\ &amp;\quad + \dfrac{\alpha_2}{2} \int_0^T\int_{\partial\Omega_a}\left(z^1_\delta -z^2_\delta\right)^2dsdt + \dfrac{w}{2} \int_0^T \int_{\Omega} \left(z^1_\delta-z^2_\delta\right)^2dxdt.\end{align*}

Using the continuity of the trace and normal trace operators, we have

\begin{equation*} J^\delta\left(\eta^*, \tau^*\right) \unicode{x2A7D} \left( \frac{\alpha_1+\alpha_2+w}{2}\right) \|z^1_\delta - z^2_\delta\|_{H^2\left(\Omega\right)}^2 \unicode{x2A7D} \left( \alpha_1+\alpha_2+w \right) \left[\|z^1_\delta\|_{H^1\left(\Omega\right)}^2 +\| z^2_\delta\|_{H^1\left(\Omega\right)}^2\right].\end{equation*}

From regularity estimates for $ z^1_\delta $ and $ z^2_\delta $, we have

\begin{equation*} \|z^1_\delta\|_{H^1\left(\Omega\right)} \unicode{x2A7D} C \|p_d^\delta - p_d\|_{H^{1/2}\left(\partial\Omega_a\right)}, \quad \|z^2_\delta\|_{H^1\left(\Omega\right)} \unicode{x2A7D} C \|p_n^\delta - p_n\|_{H^{-1/2}\left(\partial\Omega_a\right)}.\end{equation*}

and using the condition $ \|p_d^\delta-p_d\|_{H^{1/2}(\partial\Omega_a)}^2 + \|p_n^\delta-p_n\|_{H^{-1/2}(\partial\Omega_a)}^2~\unicode{x2A7D} \delta^2 $, it follows that

\begin{equation*} J^\delta\left(\eta^*, \tau^*\right) \unicode{x2A7D} \left( \alpha_1+\alpha_2+w \right) \delta^2.\end{equation*}

Since the Nash equilibrium $ (\eta_\delta^*, \tau_\delta^*) $ minimizes $ J^\delta(\eta, \tau) $, we have

\begin{equation*} J^\delta\left(\eta_\delta^*, \tau_\delta^*\right) \unicode{x2A7D} J^\delta\left(\eta^*, \tau^*\right) \unicode{x2A7D} \left( \alpha_1+\alpha_2+w \right) \delta^2,\end{equation*}

which implies

\begin{equation*} J_1^\delta\left(\eta_\delta^*, \tau_\delta^*\right)\unicode{x2A7D} \left( \alpha_1+\alpha_2+w \right) \delta^2,~ J_2^\delta\left(\eta_\delta^*, \tau_\delta^*\right)\unicode{x2A7D} \left( \alpha_1+\alpha_2+w \right) \delta^2.\end{equation*}

Since $ J^\delta(\eta, \tau) $ is coercive and strictly convex, $ \eta_N^\delta $ and $ \tau_N^\delta $ are uniformly bounded, and weak convergence follows. Taking the limit *δ* → 0, we obtain:

\begin{equation*} \eta_N^\delta \to \eta_C \quad \text{in } H^{-1/2}\left(\Gamma_i\right), \quad \tau_N^\delta \to \tau_C \quad \text{in } H^{1/2}\left(\Gamma_i\right),\end{equation*}

and the solutions $q_1^\delta(\eta_N^\delta) $ and $q_2^\delta(\tau_N^\delta) $ converge strongly in $C^0([0,T]; H^{1}(\Omega))$ to the Cauchy solution *q*. □

## 4. Numerical schemes

We consider the Hamiltonian function $H:\partial\Omega_i\times [E_l,E_r]\times[T_l,T_r]\times \mathbb{R}^2$ and is defined as follows

\begin{equation*} \begin{aligned} H\left(x,\eta\left(x\right), \tau\left(x\right), u^1\left(x\right), u^2\left(x\right)\right) &amp; = - c\left(x\right)\eta\left(x\right)u^1\left(x\right) + \dfrac{\partial}{\partial n}\left(c\left(x\right)u^2\left(x\right)\right)\tau\left(x\right),~ x \in \partial \Omega_i \end{aligned}\end{equation*}

where $u^1,u^2$ solve the following adjoint equations:

\begin{align*} \begin{aligned} &amp;u^1_{tt}-\Delta \left(c\left(x\right) u^1\left(x,t\right)\right) = w\left(q^1-q^2\right), ~ \mbox{in } Q,\\ &amp;u^1\left(x,0\right) = 0, ~ \mbox{in } \Omega,\\ &amp;u^1_t\left(x,0\right) = 0, ~ \mbox{in } \Omega,\\ &amp;u^1\left(x,t\right) = q^1-p_d, ~ \mbox{on } \partial\Omega_a \times \left(0,T\right),\\ &amp;\dfrac{\partial \left(cu^1\right)}{\partial n}\left(x,t\right) = 0, ~ \mbox{on } \partial\Omega_i \times \left(0,T\right). \end{aligned}\end{align*}

\begin{align*} \begin{aligned} &amp;u^2_{tt}-\Delta\left(c\left(x\right) u^2\left(x,t\right)\right) = w\left(q^2-q^1\right), ~ \mbox{in } Q,\\ &amp;u^2\left(x,0\right) = 0, ~ \mbox{in } \Omega,\\ &amp;u^2_t\left(x,0\right) = 0, ~ \mbox{in } \Omega,\\ &amp;u^2\left(x,t\right) = 0, ~ \mbox{on } \partial\Omega_i \times \left(0,T\right),\\ &amp;\dfrac{\partial \left(cu^2\right)}{\partial n}\left(x,t\right) = \dfrac{\partial q^2}{\partial n}-p_n, ~ \mbox{on } \partial\Omega_a \times \left(0,T\right). \end{aligned}\end{align*}

We have the following PMP characterization of a local minimum:

Theorem 6. Let $(\eta^*,\tau^*)$ be a minimizer of *J*. Then for $(v_1,v_2) \in [E_l,E_r]\times[T_l,T_r]$, we have

\begin{align*} \begin{aligned} &amp;H\left(x,\eta^*\left(x\right), \tau^*\left(x\right), u^{1*}\left(x\right), u^{2*}\left(x\right)\right) = \mathop{\mathrm{arg\,min}_{\left(v_1,v_2\right)}} H\left(x,v_1, v_2, u^{1*}\left(x\right), u^{2*}\right),\mbox{a.e. in }\partial\Omega_i, \end{aligned}\end{align*}

where $u^{i*},~ i = 1,2$ solve the adjoint wave equations.

### 4.1. A sequential quadratic Hamiltonian (SQH) method

The SQH method, introduced in [6], represents a recent advancement in successive approximation (SA) techniques. The guiding principle behind these methods is the minimization of the Hamilton-Pontryagin (HP) function associated with a given optimal control problem. Initially inspired by needle variations in the proof of the Pontryagin maximum principle (PMP) [26], this approach is particularly suited for various tomographic inverse problems that can be modeled as optimal control problems (e.g. see [11, 29]).

The starting point of our SQH method is the augmented HP function, which is defined as:

\begin{equation*}H_\epsilon\left(x,\eta, \tau, \tilde{\eta},\tilde{\tau},u^1, u^2\right) = H\left(x,\eta, \tau, u^1, u^2\right) + \epsilon \left[\left(\eta - \tilde{\eta}\right)^2+\left(\tau - \tilde{\tau}\right)^2\right].\end{equation*}

Here *ε* > 0 is a penalization parameter that is adaptively adjusted at each iteration of the SQH process. Specifically, *ε* is increased if a sufficient decrease in the functional *J*, defined in theorem 2, is not observed, and decreased if *J* decreases adequately. Furthermore, $\tilde{\eta},\tilde{\tau}$ represent the previous approximations of $\eta,\tau$ respectively. The purpose of the quadratic term $\epsilon \left[(\eta - \tilde{\eta})^2+(\tau - \tilde{\tau})^2\right]$ is to ensure that the pointwise minimizer of *H*_*ε*_, and thus the updates to $\eta,\tau$, remain close to the prior values $\tilde{\eta},\tilde{\tau}$, especially when *ε* is large. During a minimization sweep over all grid points, the values of the state $q^1,q^2$ and the adjoint $u^1,u^2$ obtained from the previous iteration are used and are only updated after a successful minimization step, leading to a fast and robust implementation. The SQH algorithm is outlined in the following algorithm:

Algorithm 4.1. (SQH method).

• Input: initial approx. $\eta^0,\tau^0$, max. number of iterations *k*_*max*_, tolerance *κ* > 0, *ε* > 0, *λ* > 1, *δ* > 0, and $\zeta\in\left(0,1\right)$; set $\gamma > \kappa $, $k: = 0$.
• Compute the solutions $q^1_0,q^2_0$ to the Cauchy problems (4) and (5) with $\tau = \tau^0,~\eta = \eta^0$.
• While ($k < k_{max} ~\&amp;\&amp; ~ \gamma > \kappa$ ) do
  (a) Compute the solutions $u^1_k,u^2_k$ to the adjoint problems (17) and (18) with $q^1 = q^1_k,~q^2 = q^2_k$.
  (b) Determine $\tau,\eta$ such that the following optimization problem is satisfied
    \begin{align*} H_\epsilon\left(x,\eta, \tau, \eta^k, \tau^k,u^1_k, u^2_k\right) = \min_{v_1,v_2\in \left[\eta_l,\eta_r\right]\times\left[\tau_l,\tau_r\right]} {H}_\epsilon \left(x,v_1,v_2, \eta^k, \tau^k,u^1_k, u^2_k\right),\end{align*}
    at almost all $x \in \partial\Omega_i$.
  (c) Compute the solutions $q^1_{k+1},q^2_{k+1}$ to the Cauchy problems (4) and (5) with the obtained $\tau,~\eta$.
  (d) Compute $\gamma: = \|\eta - \eta^k\|_{L^{2}(\partial\Omega_i)}^2+\|\tau - \tau^k\|_{L^{2}(\partial\Omega_i)}^2$.
  (e) If $J\left(\eta,\tau\right)-J\left(\eta^{k},\tau^{k}\right) > -\delta\gamma$, then increase *ε* with $\epsilon = \lambda \, \epsilon$ and go to Step (b), else decrease *ε* with $\epsilon = \zeta \, \epsilon$, set $\eta^{k+1} = \eta,~ \tau^{k+1} = \tau$, compute the solutions $u^1_{k+1},u^2_{k+1}$ to the adjoint problems (17) and (18) with $q^1 = q^1_{k+1},~q^2 = q^2_{k+1}$, and continue.
  (f) Set $k: = k+1$.
• end While

In Step (e) of this algorithm, if the inequality $J\left(\eta,\tau\right)-J\left(\eta^{k},\tau^{k}\right) > -\delta\gamma$ holds, it indicates that a sufficient decrease in the objective functional *J* has not been achieved. In such a case, *ε* is increased (since *λ* > 1), and the optimization in Step (b) is repeated with the updated augmented HP function. Conversely, if the inequality does not hold, it confirms that the sufficient decrease of *J* has been achieved. Then $\eta^{k+1},\tau^{k+1}$ are chosen as the desired updates, along with the corresponding updates of $q^1_{k+1},q^2_{k+1}$ and $u^1_{k+1},u^2_{k+1}$ for the wave equations and their adjoints. In this situation, *ε* is reduced by a factor *ζ* < 1.

Using similar arguments as in [11, 29], one can actually show the convergence property of the SQH scheme in the following results:

Lemma 7. Let $(\eta^k,\tau^k)$ and $(\eta^{k+1},\tau^{k+1})$ be generated by the SQH scheme, $k\in\mathbb{N}_0$. Then there exists an *ε* > 0 such that $J(\eta^{k+1},\tau^{k+1})-J(\eta^k,\tau^k) < 0.$

Theorem 8. Let the sequences $(\eta^k,\tau^k)$ be generated by the SQH scheme. Then the sequence of cost functional values $J(\eta^k,\tau^k)$ monotonically decreases with

\begin{equation*} \lim_{k\rightarrow \infty}\left[J\left(\eta^{k+1},\tau^{k+1}\right) - J\left(\eta^k,\tau^k\right)\right] = 0,\end{equation*}

and

\begin{equation*} \begin{aligned} &amp;\lim_{k\rightarrow \infty} \|\left(\eta^{k+1},\tau^{k+1}\right)-\left(\eta^k,\tau^k\right)\|_2 = 0.\\ \end{aligned}\end{equation*}

### 4.2. A PINN framework for estimating the initial guess

To solve for $\eta,\tau$ using the SQH scheme, it requires an initial guess. Traditional options are to either provide a specified initial guess, like 0, or construct an approximate solution using some other algorithm and use that as the initial guess. For specified initial guesses, iterative algorithms like SQH work better if the structure of the initial guess resembles the structure of the true solution. Even though *p_d_* might have a structure like sparsity or piecewise continuity, it is difficult to predict *p_n_*. Alternatively, one can approximate initial guesses using alternate algorithms: solve for $p_0(x)$ using a traditional iterative algorithm and simulate the forward wave equation to obtain the missing data approximation. However, this is time-consuming, computationally intensive, and lacks stability. We aim at a fast, robust, and accurate solution to the identification problem of finding an estimate for *H*, given Cauchy data, using PINN, i.e. Find *p*_0_ such that *p* is close to $p_d,p_n$, with *p* satisfying

\begin{equation*} \begin{aligned} &amp;p_{tt}-c\left(x\right)\Delta p\left(x,t\right) = 0, ~ \mbox{in } \Omega \times \left(0,T\right),\\ &amp;p\left(x,0\right) = p_0\left(x\right), ~ \mbox{in } \Omega,\\ &amp;p_t\left(x,0\right) = 0, ~ \mbox{in } \Omega,\\ &amp;p\left(x,t\right) = {p_d\left(x,t\right)}, ~ \mbox{on } \partial\Omega_a \times \left(0,T\right),\\ &amp;\dfrac{\partial p}{\partial n}\left(x,t\right) = {p_n\left(x,t\right)},~ \mbox{on } \partial\Omega_a \times \left(0,T\right).\\ \end{aligned}\end{equation*}

In this approach, the solution $f(x, t)$ of the wave equation (4.2) is approximated by a neural network, denoted as $p_{NN}(x, t; p_0)$, which takes the coordinates (*x*, *t*) as inputs and generates an estimate of *u* at these points. The network *p*_*NN*_ is characterized by parameters *φ*, representing its weights and biases, along with the unknown initial condition *H*, both of which must be trained. The inputs are processed through a series of dense layers *l*, forming the architecture of *p*_*NN*_ as follows:

1. Input layer: $w^0 = (x, t)$
2. *l* hidden layers: $w^k = \sigma(\Phi_W^k \cdot w^{k-1} + \Phi_B^k)$ for $1 \unicode{x2A7D} k \unicode{x2A7D} l-1$
3. Output layer: $w^l = \Phi_W^l \cdot w^{l-1} + \Phi_B^l$.

In each layer *k*, an output vector $w^k \in \mathbb{R}^{r_k}$ is produced, where *r_k_* represents the number of neurons in that layer. The transformations within the layer are governed by weight matrices $\Phi_W^k$ and bias vectors $\Phi_B^k$, combined with a nonlinear activation function *σ*, which is applied at each hidden layer. The final output *w^l^* serves as the approximate solution $p_\mathrm{NN}(x, t; p_0)$. Here, the set of network parameters to be trained is given by $\Phi = (\Phi_W, \Phi_B) = \phi \cup p_0$.

A key advantage of PINNs is their ability to integrate the dynamical structure of the system during training, allowing the network to learn a physics-informed mapping without relying on extensive datasets. This is achieved by incorporating a modified loss function that accounts for discrepancies in the physics described by the wave equation (4.2), as well as the initial conditions. The physics-based loss function is defined as follows:

\begin{equation*} \begin{aligned} \mathcal{L}_P\left(\phi, H\right) &amp; = \frac{w_P}{N_p} \sum_{i = 1}^{N_p} \left[ \mathcal{E}\left(p_\mathrm{NN}\left(\boldsymbol{x}_i^p, t_i^p\right), p_0\right) \right]^2\\ &amp;\quad + \frac{w_0}{N_0} \sum_{i = 1}^{N_0} \left[ p_\mathrm{NN}\left(\boldsymbol{x}_i^0, 0\right)-p_0\left(\boldsymbol{x}_i^0, 0\right) \right]^2 + \frac{w_0^t}{N_0} \sum_{i = 1}^{N_0} \left[ \partial_tp_\mathrm{NN}\left(\boldsymbol{x}_i^0, 0\right) \right]^2 , \end{aligned}\end{equation*}

where $(\boldsymbol{x}_i^p, t_i^p)_{i = 1}^{N_p}$ and $(\boldsymbol{x}_i^0)_{i = 1}^{N_0}$, represent sets of points selected to enforce the wave equation (4.2) and the initial conditions, respectively. The parameters $w_p, w_0$ control the contributions of each loss component. To determine the *p*_0_, the loss function $\mathcal{L}_P$ can be augmented with a regularized data loss function given below:

\begin{equation*} \mathcal{L}_D\left(\phi, p_0\right) = \dfrac{w_B^1}{2N_b} \sum_{i = 1}^{N_b} \left[ p_\mathrm{NN}\left(\boldsymbol{x}_i^b, t_i^b\right) - p_d\left(\boldsymbol{x}_i^d, t_i^d\right) \right]^2 + \dfrac{w_B^2}{2N_b} \sum_{i = 1}^{N_b} \left[ \partial_np_\mathrm{NN}\left(\boldsymbol{x}_i^b, t_i^b\right) - p_n\left(\boldsymbol{x}_i^b, t_i^b\right) \right]^2,\end{equation*}

where $(\boldsymbol{x}_i^b, t_i^b)_{i = 1}^{N_b}$ are the set of points on the accessible boundary $\partial\Omega_a$. Thus, the combined total loss function to solve for $\boldsymbol{\theta}_u^*$ is given as

\begin{align*} \mathcal{L}\left(\phi, \boldsymbol{\theta}_u\right) = \mathcal{L}_P\left(\phi, \boldsymbol{\theta}_u\right) + \mathcal{L}_D\left(\phi, \boldsymbol{\theta}_u\right).\end{align*}

The NN parameters $\Phi = \phi \cup p_0$ are iteratively optimized to find $(\phi^*,p_0^*)$ that leads to the solution and the Neumann derivative of the wave equation $(4.2)$ approximately close to $p_d,p_n$, respectively, at $\partial\Omega_a$. At each iteration *k*, the update rule for the NN parameters is:

\begin{equation*} \Phi^{k+1} = \Phi^k - \alpha\left(k\right) \nabla_{\Phi} \mathcal{L}\left(\Phi^k\right),\end{equation*}

where $\alpha(k)$ is an adaptive learning rate. The trained network $p_\mathrm{NN}(x, t; \phi^*, p_0^*)$ then provides an estimate of *p*_0_, which is used to obtain an initial guess of $\eta,\tau$ for the minimization problem (9).

## 5. Results

In this section, we present the results of our PINN-SQH method to solve the NASH GAME problem to obtain the initial pressure *H* from limited Cauchy data. For generating the initial guess using a PINN, we use an architecture with 8 dense layers for the wave PDE and 4 dense layers for the initial condition with 64 neurons in each layer. We also used the the hyperbolic tangent (tanh) activation function. This configuration was selected after testing several alternatives (ranging from 3–10 layers and 20–100 neurons per layer) and observing the trade-off between approximation accuracy and training stability. The tanh activation ensures smoothness of the solution and facilitates the representation of the continuous spatial-temporal fields involved in the inverse wave problem. Furthermore, for the loss function, relative weights were empirically tuned through preliminary numerical experiments to ensure that all loss components are of comparable magnitude during training. Specifically, the boundary and initial condition losses were assigned weights one order of magnitude lower than the PDE residual term to promote physical consistency in the domain. The final choice of weights was found to provide stable convergence without overshooting or imbalance among the competing terms. To circumvent potential convergence to local minima, we employed multiple strategies: (i) initialization with small random weights following the Xavier scheme, and (ii) an adaptive learning rate schedule, starting from 0.001, and decaying by a factor of 0.9 every 1000 iterations. The training is done on an uniform set of grid points in the space-time domain, with 5000 epochs to achieve an $l^2_2-$norm loss of the order 10^−3^. The parameters for the SQH algorithm are chosen as: $\kappa = 9e-10,~ \epsilon = 1,~ \lambda = 1.1,~ \zeta = 0.9,~ \delta = 1e-6.$

We also compare the reconstructions obtained with our PINN-SQH algorithm to the reconstructions obtained with the traditional TR algorithm, the PINN algorithm, and the SQH algorithm without the initial guess obtained from the PINN algorithm. For qualitative comparison, we also use the quantitative figure of merits: Mean-Square Error (MSE) and the Peak Signal-to-Noise Ratio (PSNR) defined below:

\begin{equation*} \mathrm{MSE}\left(I^1,I^2\right) = \dfrac{1}{n}\sum_{p = 1}^n \left(I_p^1-I^2_p\right)^2,~ \mathrm{PSNR}\left(I^1,I^2\right) = 10\log_{10}\left(\dfrac{\left[\max\left({I^1,I^2}\right)\right]^2}{\mathrm{MSE}\left(I^1,I^2\right)}\right),\end{equation*}

where *p* represents a pixel and *n* is the total number of pixels.

**Figure 1.**
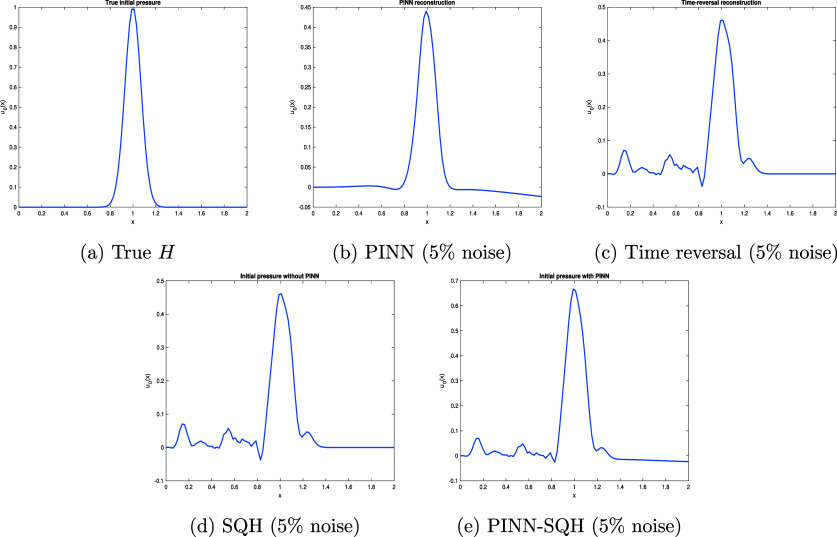
Test Case 1(a): Reconstructions of the 1D Gaussian phantom with 5% additive Gaussian noise.

We first present the results in a one-dimensional setup. For this purpose, we choose our domain $\Omega = (0,2)$ and the final time of observations as *T* = 1.5. The complete observation domain $\partial\Omega = x = \lbrace0,2\rbrace$ while our accessible observation domain is given as $\partial\Omega_a = x = \lbrace0\rbrace$. For the true *H*, we consider a Gaussian phantom centered at *x* = 1 with a peak value of 1.0 and standard deviation 0.25. We also choose a non-trapping sound speed $c(x) = 1+w(x)*0.1*\cos(2\pi x)$, where *w*(*x*) is a mollifier centered at the middle of the domain with radius $\sqrt{0.5}$, achieving a maximum value of 1. For the spatial grid, we choose 100 points, whereas our time grid comprises of 100 points. To generate the data, we solve the wave equation (1) in free space on the same spatial grid but at only 20 time points, and then interpolate the solution on the time grid to obtain $p_d,p_n$ at *x* = 0. Such a setup is conforming to the ranges used for the number of sensors in a photoacoustic machine with limited sampling frequency (see e.g. [34].) Figure 1 shows the results of the reconstructions with 5% additive Gaussian noise in the data.

Figure 1(a) shows the true phantom for the initial pressure. Figure 1(b) shows the reconstruction using the PINN algorithm. Figure 1(c) shows the reconstruction with the TR algorithm. Figure 1(d) shows the reconstruction with the SQH algorithm without the PINN-generated initial guess. Figure 1(e) shows the reconstruction with our PINN-SQH algorithm. We observe that the PINN reconstruction is free from a lot of artifacts but lacks contrast. The reconstructions with the TR and SQH algorithm exhibit significant artifacts and loss of contrast. On contrary, our PINN-SQH algorithm leads to reduction of artifacts and improvement of contrast.

**Figure 2.**
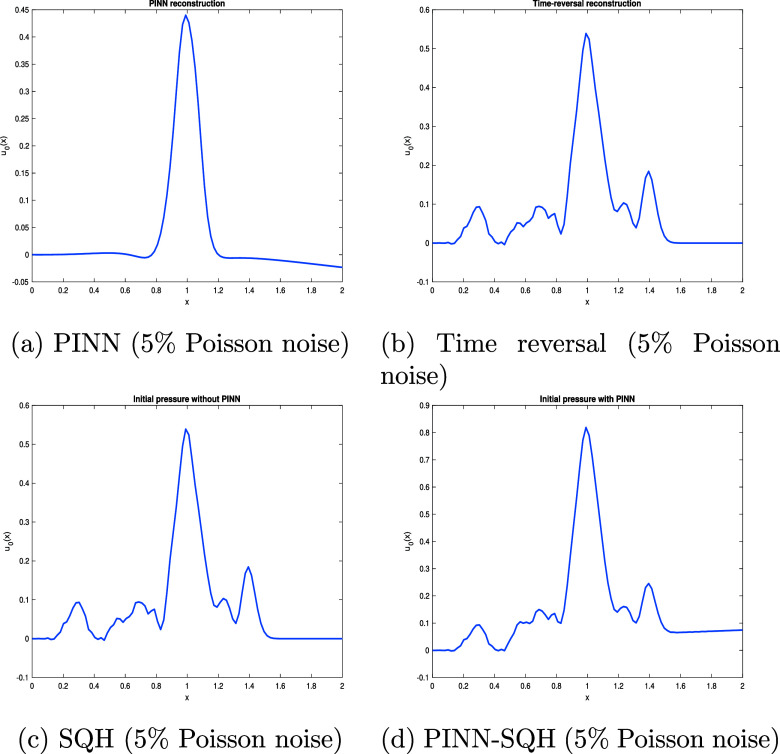
Test Case 1(b): Reconstructions of the 1D Gaussian phantom with 5% additive Poisson noise.

In the next case, we have a similar setup as above but with 5% additive Poisson noise in the data. The results are shown in figure 2.

We again observe that our PINN-SQH algorithm provides superior resolution and contrast images compared to the other methods.

With the observations made from the one-dimensional case about the superior performance of the PINN-SQH algorithm, we now proceed to the numerical results of our framework in the two-dimensional setup. Our domain $\Omega = (-1,1)\times(-1,1)$ with the complete observation domain as $\lbrace{-1,1\rbrace}\times (-1,1) \cup (-1,1)\times\lbrace{-1,1\rbrace}$. The final time of observations is *T* = 4.0. The sound speed is chosen as $c(x) = 1+w(x)*[0.1*\cos(2\pi x_1) + 0.05*\sin(2\pi x_2)],$ where *w*(*x*) is a mollifier centered at the middle of the domain with radius $\sqrt{0.5}$, with a maximum value of 1.

**Figure 3.**
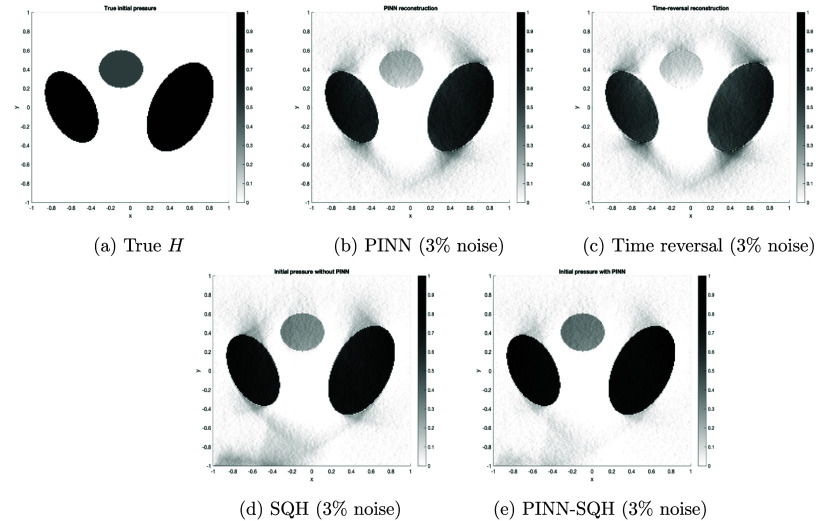
Test Case 2(a): Reconstructions of the heart and lung phantom with 3% additive Gaussian noise.

**Figure 4.**
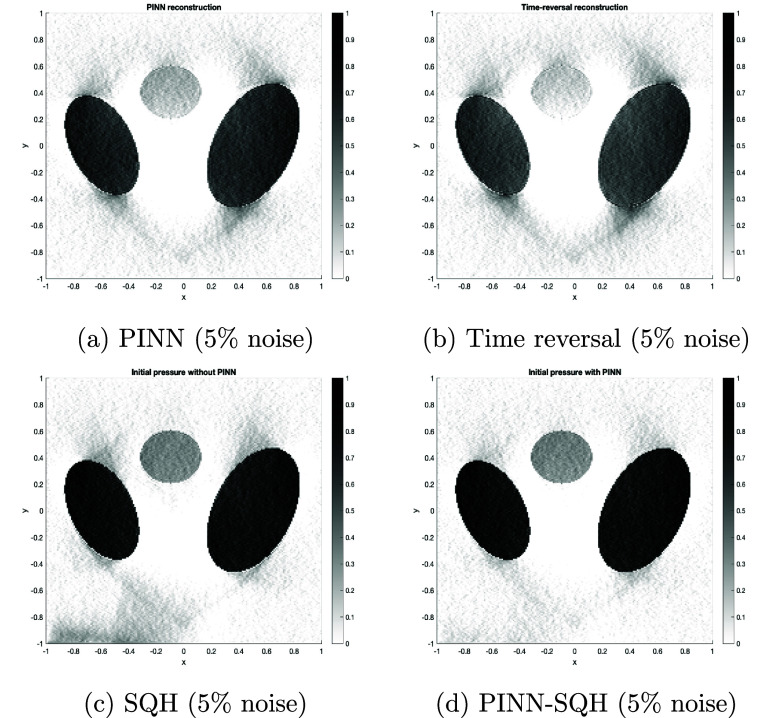
Test Case 2(b): Reconstructions of the heart and lung phantom with 5% additive Gaussian noise.

**Figure 5.**
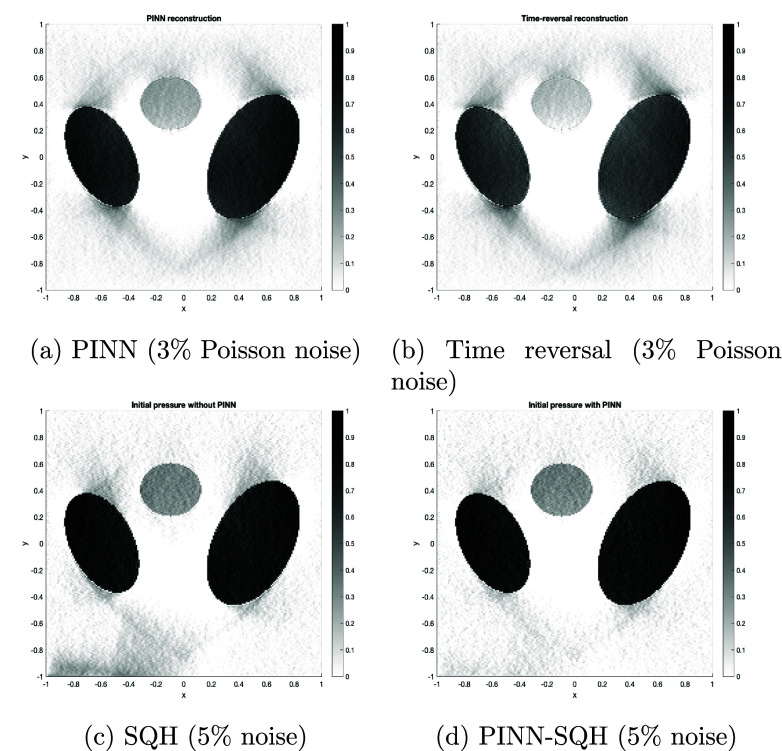
Test Case 2(c): Reconstructions of the heart and lung phantom with 3% additive Poisson noise.

**Figure 6.**
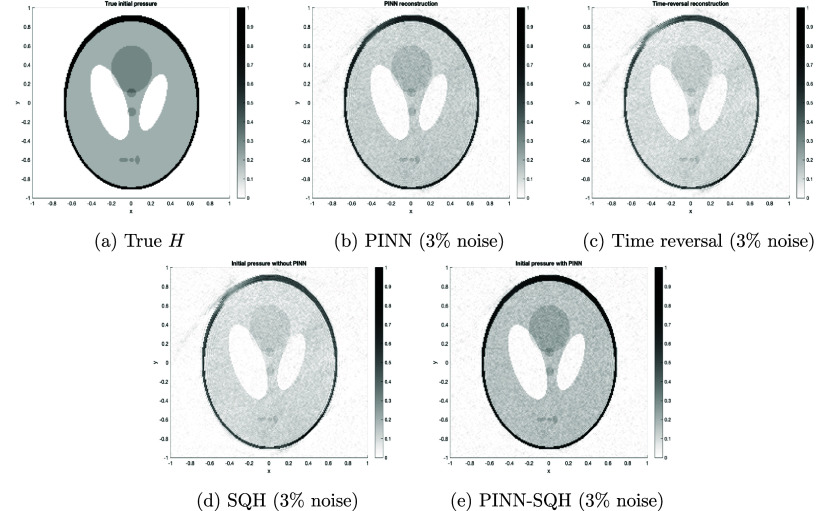
Test Case 3(a): Reconstructions of the Shepp-Logan phantom with 3% additive Gaussian noise.

**Figure 7.**
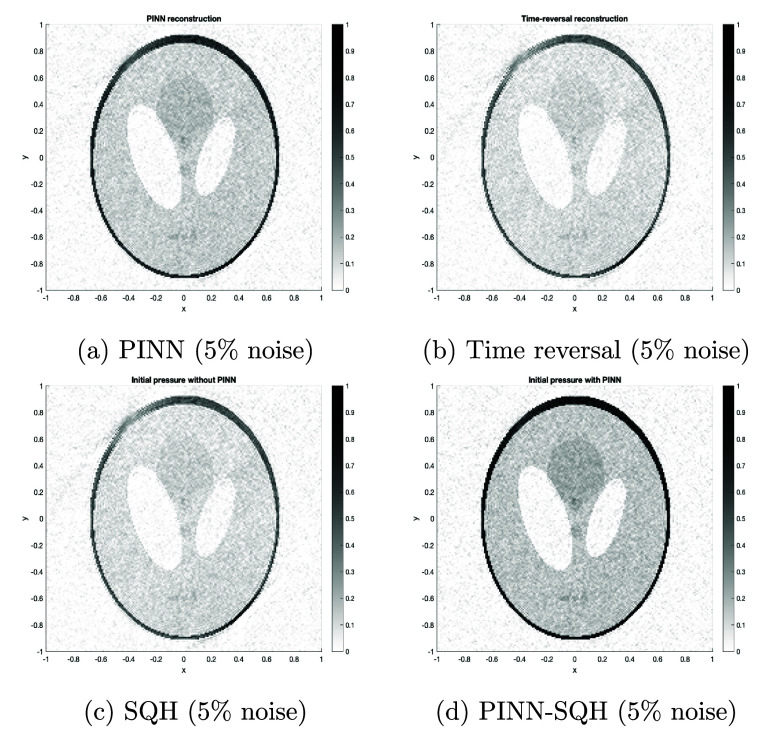
Test Case 3(b): Reconstructions of the Shepp-Logan phantom with 5% additive Gaussian noise.

**Figure 8.**
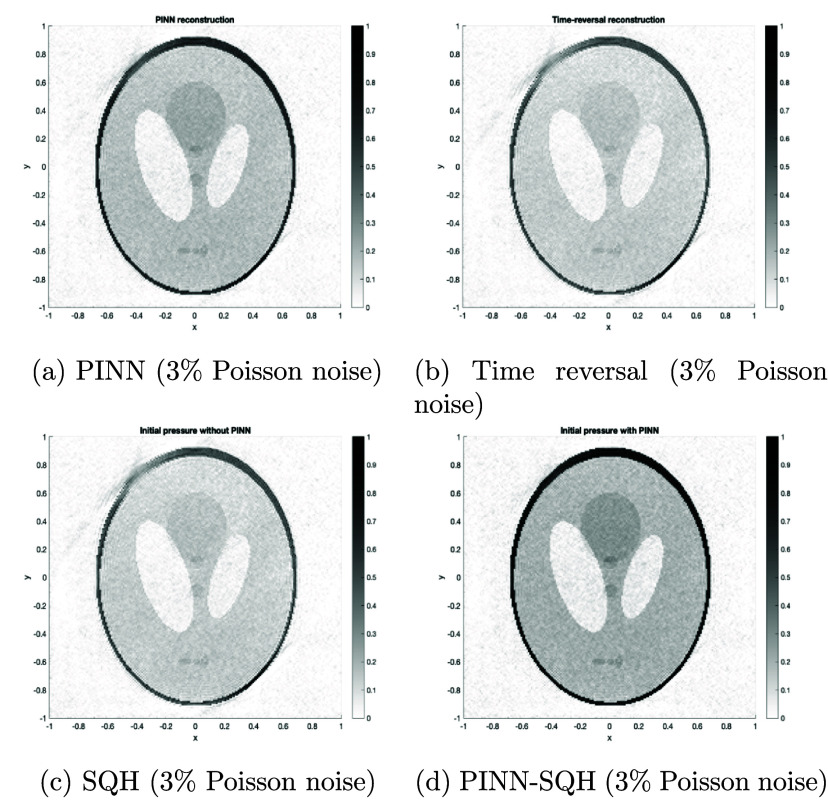
Test Case 3(c): Reconstructions of the Shepp-Logan phantom with 3% additive Poisson noise.

**Figure 9.**
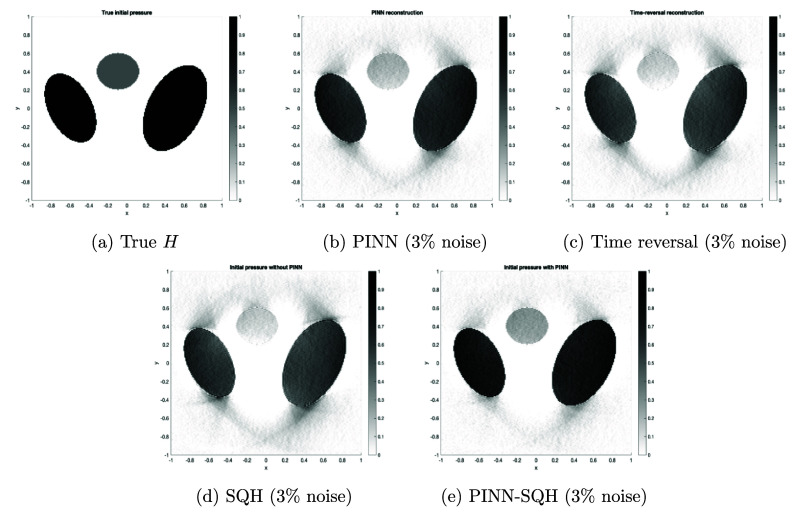
Test Case 4(a): Reconstructions of the heart and lung phantom with 3% additive Gaussian noise.

**Figure 10.**
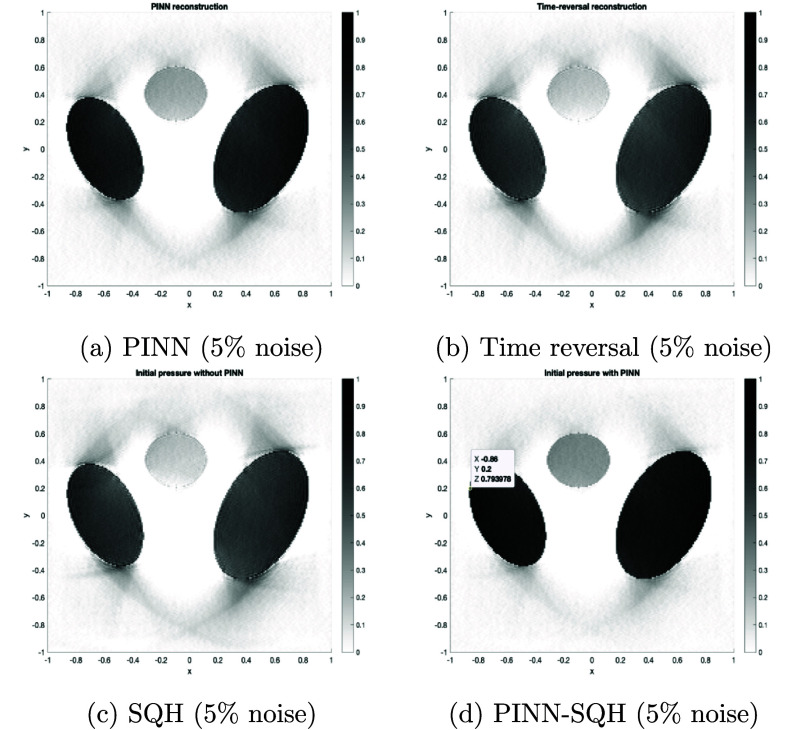
Test Case 4(b): Reconstructions of the heart and lung phantom with 5% additive Gaussian noise.

**Figure 11.**
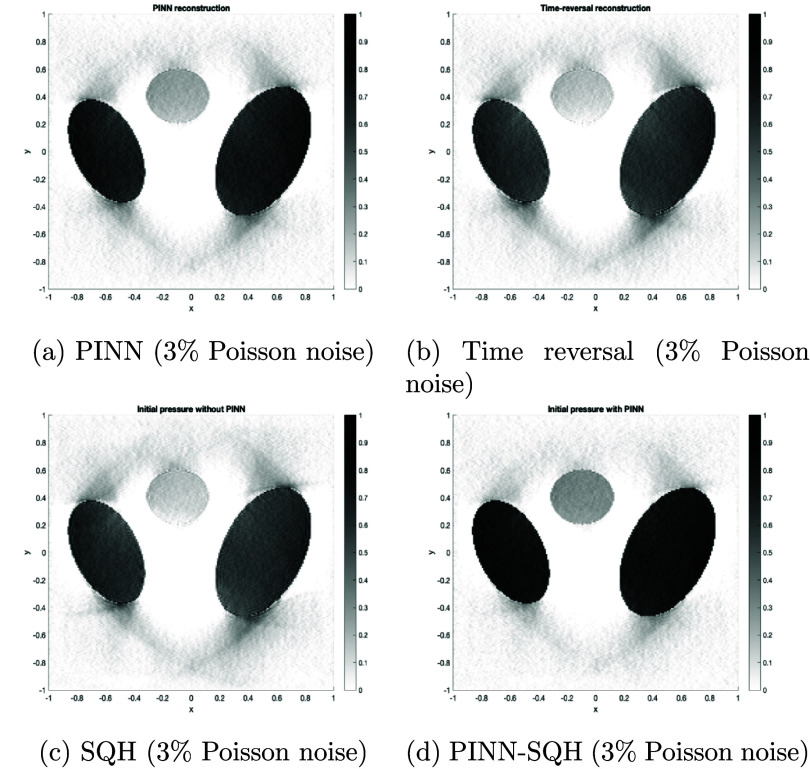
Test Case 4(c): Reconstructions of the heart and lung phantom with 3% additive Poisson noise.

**Figure 12.**
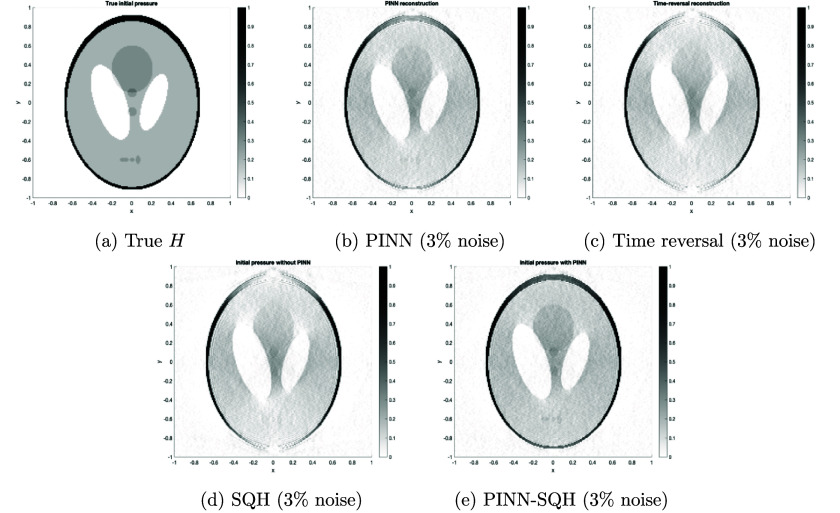
Test Case 5(a): Reconstructions of the Shepp-Logan phantom with 3% additive Gaussian noise.

**Figure 13.**
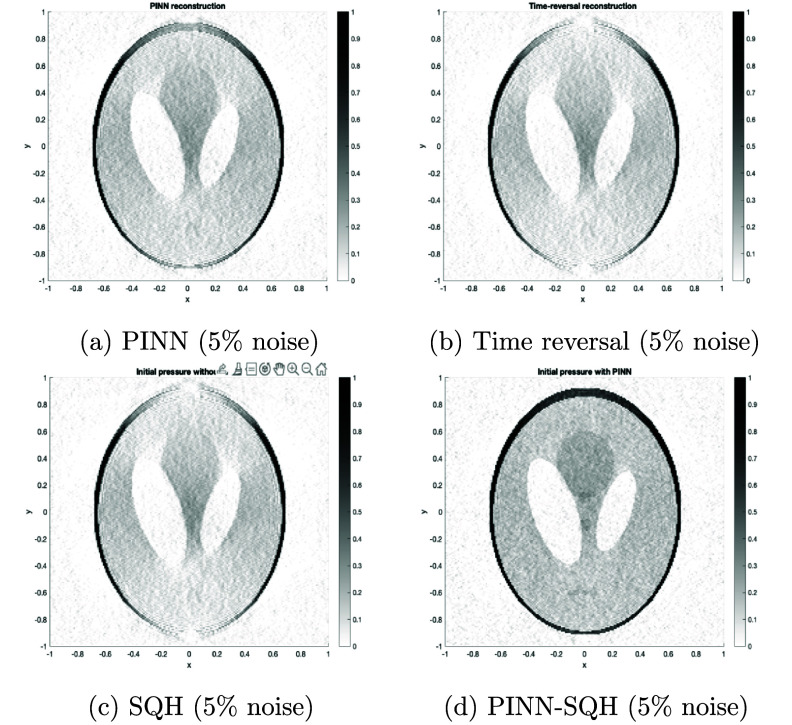
Test Case 5(b): Reconstructions of the Shepp-Logan phantom with 5% additive Gaussian noise.

**Figure 14.**
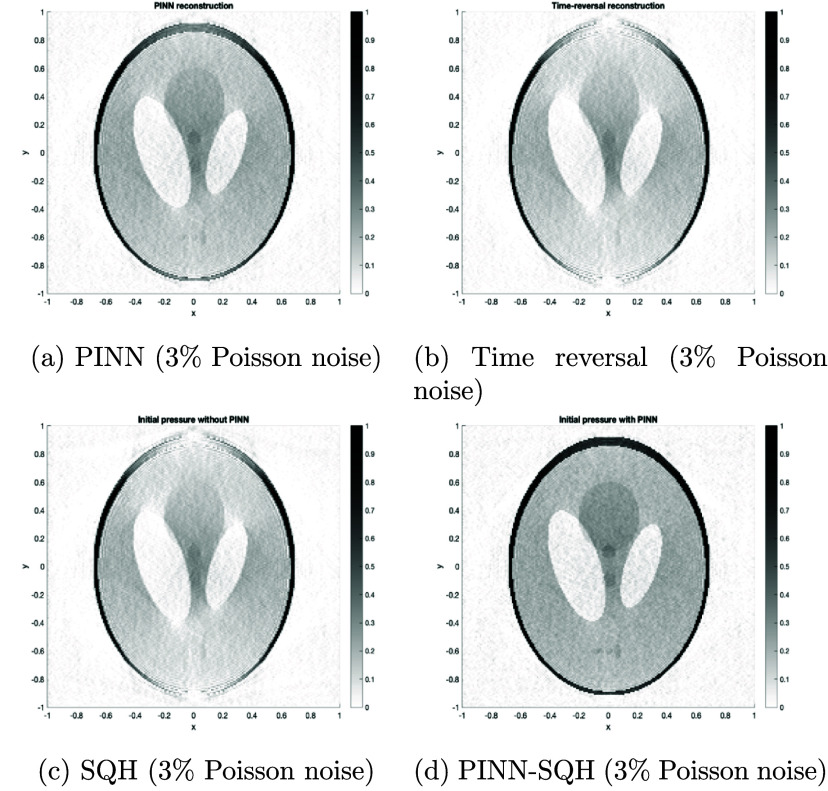
Test Case 5(c): Reconstructions of the Shepp-Logan phantom with 3% additive Poisson noise.

1. **Accessible observation domain- $\lbrace{-1\rbrace}\times (-1,1) \cup (-1,1) \times \lbrace -1 \rbrace:$**
  In our first set of test cases, we consider the accessible observation domain to be the left and the bottom boundary of the square domain Ω. This mimics the scenario when the ultrasonic transducers are placed along a semi-circular one side of the patient and the other side is inaccessible.
  We now consider the subcase simulated with a heart and lung phantom. It consists of two ellipses representing lungs with intensity 1.0 and a circular region representing the heart with intensity 0.5. Figure 3 shows the reconstructions using various methods with 3% additive Gaussian noise in the data.
  Figure 3(b) shows the reconstruction with the PINN algorithm, figure 3(c) shows the reconstruction with the TR algorithm, figure 3(d), shows the reconstruction with the SQH algorithm but with a zero initial guess, and figure 3(e) shows the reconstruction with the SQH method. We note that there is a significant loss of contrast and resolution with the TR algorithm and the SQH algorithm with a zero initial guess. The PINN reconstruction improve in contrast and resolution but the best resolution and contrast is achieved with the PINN-SQH algorithm.
  In our next subcase, we consider the same set of reconstructions as above, but now with 5% additive Gaussian noise. The reconstructions are shown in figure 4.
  We again that there is a significant loss of contrast and resolution with the TR algorithm and the SQH algorithm with a zero initial guess. The PINN reconstruction improve in contrast and resolution but the best resolution and contrast is achieved with the PINN-SQH algorithm.
  In our next subcase, we consider the same set of reconstructions as above, but now with 3% additive Poisson noise. The reconstructions are shown in figure 5.
  Even with a different type of noise patter, we again observe that there is a significant loss of contrast and resolution with the TR algorithm and the SQH algorithm with a zero initial guess. The PINN reconstruction improve in contrast and resolution but the best resolution and contrast is achieved with the PINN-SQH algorithm.
  In our next set of subcases, we consider our initial pressure phantom as the Shepp-Logan phantom. We again consider the reconstructions with 3% additive Gaussian noise in the data as shown in figure 6.
  We note that there is a significant loss of contrast and resolution with the TR algorithm and the SQH algorithm with a zero initial guess. Notably, the 3 small ellipses have the least contrast making these algorithms inept at reconstructing fine structures with limited and noisy data. The PINN reconstruction improve in contrast and resolution and with the PINN-SQH algorithm we get the best reconstruction, with even the small ellipses having good contrast.
  In our next subcase, we consider the same set of reconstructions as above, but now with 5% additive Gaussian noise. The reconstructions are shown in figure 7.
  We again observe that there is a significant loss of contrast and resolution with the TR algorithm and the SQH algorithm with a zero initial guess. The PINN reconstruction improve in contrast and resolution but the best resolution and contrast is achieved with the PINN-SQH algorithm.
  In our next subcase, we consider the same set of reconstructions as above, but now with 3% additive Poisson noise. The reconstructions are shown in figure 8.
  Again, we observe that there is a significant loss of contrast and resolution with the TR algorithm and the SQH algorithm with a zero initial guess. The PINN reconstruction improve in contrast and resolution but the best resolution and contrast is achieved with the PINN-SQH algorithm.
2. **Accessible observation domain- $\lbrace{-1,1\rbrace}\times (-1,1):$**
  In our second set of test cases, we consider the accessible observation domain to be the top and the bottom boundary of the square domain Ω. This mimics the scenario when the ultrasonic transducers are placed along two secluded zones and the remaining zones are inaccessible.
  We now consider the subcase simulated with a heart and lung phantom. It consists of two ellipses representing lungs with intensity 1.0 and a circular region representing the heart with intensity 0.5. Figure 9 shows the reconstructions using various methods with 3% additive Gaussian noise in the data.
  We note that there is a significant loss of contrast and resolution with the TR algorithm and the SQH algorithm with a zero initial guess. Specifically, due to the top and the bottom boundaries being inaccessible, there are a lot of artifacts on the object boundaries with wavefronts along those two directions. Such a problem disappears with the PINN reconstruction, resulting in improvement of contrast and resolution. The reconstruction is further enhanced with the PINN-SQH algorithm.
  In our next subcase, we consider the same set of reconstructions as above, but now with 5% additive Gaussian noise. The reconstructions are shown in figure 10.
  We again note that there is a significant loss of contrast and resolution with the TR algorithm and the SQH algorithm with a zero initial guess. The PINN reconstruction improve in contrast and resolution but the best resolution and contrast is achieved with the PINN-SQH algorithm.
  In our next subcase, we consider the same set of reconstructions as above, but now with 3% additive Poisson noise. The reconstructions are shown in figure 11.
  As before, we observe that there is a significant loss of contrast and resolution with the TR algorithm and the SQH algorithm with a zero initial guess. The PINN reconstruction improve in contrast and resolution but the best resolution and contrast is achieved with the PINN-SQH algorithm. In our next set of subcases, we consider our initial pressure phantom as the Shepp-Logan phantom. We again consider the reconstructions with 3% additive Gaussian noise in the data as shown in figure 12.
  We note that there is a significant loss of contrast and resolution with the TR algorithm and the SQH algorithm with a zero initial guess. Specifically, due to the top and the bottom boundaries being inaccessible, there are a lot of artifacts on the object boundaries with wavefronts along those two directions. The small ellipses also disappear since they are close to the bottom inaccessible boundary. With the PINN reconstruction, we have some improvement of contrast and resolution, but the small ellipses are barely visible. Also, there is still some loss of resolution near the top and the bottom inaccessible boundaries. The reconstruction gets significantly better with the PINN-SQH algorithm, where we can also observe the small ellipses.
  In our next subcase, we consider the same set of reconstructions as above, but now with 5% additive Gaussian noise. The reconstructions are shown in figure 13.
  We again note that there is a significant loss of contrast and resolution with the TR algorithm and the SQH algorithm with a zero initial guess. The PINN reconstruction results in improvement of contrast and resolution. The reconstruction is further enhanced with the PINN-SQH algorithm.
  In our next subcase, we consider the same set of reconstructions as above, but now with 3% additive Poisson noise. The reconstructions are shown in figure 14.
  As with the previous case, we again observe that there is a significant loss of contrast and resolution with the TR algorithm and the SQH algorithm with a zero initial guess. The PINN reconstruction results in improvement of contrast and resolution. The reconstruction is further enhanced with the PINN-SQH algorithm.

We now list the MSE and PSNR values of the reconstructions in tables 1 and 2.

**Table 1.** MSE values for the various test cases.

Phantom	Test Case	Noise	TR	PINN	SQH	PINN-SQH
1D Gaussian	Test case 1(a)	Gaussian ($5\%)$	0.0168	0.0186	0.1649	0.0061
1D Gaussian	Test case 1(b)	Poisson ($5\%)$	0.0156	0.0186	0.1894	0.0098
Heart-Lung	Test case 2(a)	Gaussian ($3\%)$	0.0543	0.0268	0.0547	0.0093
Heart-Lung	Test case 2(b)	Gaussian ($5\%)$	0.0556	0.0283	0.0559	0.011
Heart-Lung	Test case 2(c)	Poisson ($3\%)$	0.0479	0.0216	0.0484	0.0066
Shepp-Logan	Test case 3(a)	Gaussian ($3\%)$	0.0213	0.0109	0.0209	0.0043
Shepp-Logan	Test case 3(b)	Gaussian ($5\%)$	0.0226	0.0123	0.0222	0.006
Shepp-Logan	Test case 3(c)	Poisson ($3\%)$	0.0194	0.0096	0.0191	0.0041
Heart-Lung	Test case 4(a)	Gaussian ($3\%)$	0.0598	0.0295	0.0594	0.0102
Heart-Lung	Test case 4(b)	Gaussian ($5\%)$	0.0611	0.0310	0.0608	0.0120
Heart-Lung	Test case 4(c)	Poisson ($3\%)$	0.0548	0.0254	0.0541	0.0081
Shepp-Logan	Test case 5(a)	Gaussian ($3\%)$	0.0185	0.0096	0.0182	0.004
Shepp-Logan	Test case 5(b)	Gaussian ($5\%)$	0.0197	0.0109	0.0192	0.0057
Shepp-Logan	Test case 5(c)	Poisson ($3\%)$	0.0161	0.0078	0.0161	0.0035

**Table 2.** PSNR values for the various test cases.

Phantom	Test Case	Noise	TR	PINN	SQH	PINN-SQH
1D Gaussian	Test case 1(a)	Gaussian ($5\%)$	17.76	17.31	7.83	22.13
1D Gaussian	Test case 1(b)	Poisson ($5\%)$	18.07	17.31	7.23	20.11
Heart-Lung	Test case 2(a)	Gaussian ($3\%)$	12.65	15.72	12.62	20.31
Heart-Lung	Test case 2(b)	Gaussian ($5\%)$	12.55	15.48	12.53	19.58
Heart-Lung	Test case 2(c)	Poisson ($3\%)$	13.20	16.65	13.15	21.80
Shepp-Logan	Test case 3(a)	Gaussian ($3\%)$	16.72	19.63	16.81	23.68
Shepp-Logan	Test case 3(b)	Gaussian ($5\%)$	16.45	19.09	16.54	22.22
Shepp-Logan	Test case 3(c)	Poisson ($3\%)$	17.11	20.17	17.18	23.83
Heart-Lung	Test case 4(a)	Gaussian ($3\%)$	12.23	15.30	12.26	19.90
Heart-Lung	Test case 4(b)	Gaussian ($5\%)$	12.14	15.09	12.18	19.21
Heart-Lung	Test case 4(c)	Poisson ($3\%)$	12.61	15.95	12.65	20.91
Shepp-Logan	Test case 5(a)	Gaussian ($3\%)$	17.34	20.20	17.41	24.00
Shepp-Logan	Test case 5(b)	Gaussian ($5\%)$	17.05	19.61	17.10	22.44
Shepp-Logan	Test case 5(c)	Poisson ($3\%)$	17.94	21.09	17.94	24.55

We observe that the TR and the SQH method without the PINN initial guess gives the highest MSE and the lowest PSNR values. The MSE decreases and PSNR increases with the PINN reconstruction but they are significantly better with the PINN-SQH algorithm, further demonstrating the robustness and versatility of our proposed framework.

## 6. Conclusions

This paper introduces a novel and effective methodological framework designed to achieve superior image reconstructions in the context of limited-view PAT, where traditional reconstruction techniques often suffer from degraded resolution and artifacts due to incomplete boundary measurements. The core idea of the proposed framework is to leverage the available Cauchy data i.e. the combination of pressure and its normal derivative on an accessible portion of the observation domain, and to infer the missing data on the inaccessible part using a game-theoretic approach. Specifically, we formulate this data completion problem as a two-player Nash equilibrium game, where each player controls the boundary data on a subdomain and seeks to minimize a local cost functional. This setting naturally enforces consistency of the reconstructed data across the accessible and inaccessible regions. To solve the resulting Nash equilibrium problem efficiently, we employ a gradient-free SQH method, which is rooted in Pontryagin’s maximum principle and well-suited for handling the non-smoothness and high dimensionality of the control space. This is coupled with PINNs, which are used to construct a physics-consistent initial guess by embedding the underlying photoacoustic wave equation into the training process. The PINN-based initialization ensures that the SQH scheme starts from a feasible and physically meaningful state, thereby improving convergence and stability of the iterative process. Numerical simulations are conducted using several standard phantom models to validate the performance of the proposed hybrid framework. The results demonstrate that the method yields high-contrast and high-resolution reconstructions, even under severe data limitations. Compared to existing approaches, our method significantly reduces artifacts and preserves fine structural details, underscoring the advantages of combining game-theoretic data completion with physics-based deep learning techniques for solving ill-posed inverse problems in photoacoustic tomography.

## Data Availability

No new data were created or analysed in this study.
